# Clinicopathological and Molecular Prognostic Classifier for Intermediate/High-Risk Clear Cell Renal Cell Carcinoma

**DOI:** 10.3390/cancers13246338

**Published:** 2021-12-17

**Authors:** Fiorella L. Roldán, Juan J. Lozano, Mercedes Ingelmo-Torres, Raquel Carrasco, Esther Díaz, Miguel Ramirez-Backhaus, José Rubio, Oscar Reig, Antonio Alcaraz, Lourdes Mengual, Laura Izquierdo

**Affiliations:** 1 Department and Laboratory of Urology, Hospital Clínic, Institut d’Investigacions Biomèdiques August Pi i Sunyer (IDIBAPS), Universitat de Barcelona, 08036 Barcelona, Spain; flroldan@clinic.cat (F.L.R.); ingelmo@clinic.cat (M.I.-T.); rcarrasco@clinic.cat (R.C.); ediazs@clinic.cat (E.D.); aalcaraz@clinic.cat (A.A.); lizquier@clinic.cat (L.I.); 2 Bioinformatics Platform, Centro de Investigación Biomédica en Red de Enfermedades Hepáticas y Digestivas (CIBERehd), Hospital Clinic, 08036 Barcelona, Spain; juanjo.lozano@ciberehd.org; 3 Department of Urology, Oncologic Institute of Valencia, 46009 Valencia, Spain; miramirez@fivo.org (M.R.-B.); jrubio@fivo.org (J.R.); 4 Translational Genomics and Targeted Therapeutics in Solid Tumors, August Pi i Sunyer Biomedical Research Institute (IDIBAPS) and Medical Oncology Department, Hospital Clínic de Barcelona, 08036 Barcelona, Spain; OREIG@clinic.cat; 5 Department of Biomedical Sciences, Faculty of Medicine and Health Sciences, University of Barcelona, 08036 Barcelona, Spain

**Keywords:** gene expression, clear cell renal cell carcinoma, disease progression, prognostic factors, biomarkers, RNA sequencing

## Abstract

**Simple Summary:**

In this report, we identified biomarkers for tumor progression from tissue samples of intermediate/high-risk ccRCC. Using the molecular findings and the clinical data, we developed an improved prognostic model which could help to provide better individualized management recommendations.

**Abstract:**

The probability of tumor progression in intermediate/high-risk clear cell renal cell carcinoma (ccRCC) is highly variable, underlining the lack of predictive accuracy of the current clinicopathological factors. To develop an accurate prognostic classifier for these patients, we analyzed global gene expression patterns in 13 tissue samples from progressive and non-progressive ccRCC using Illumina Hi-seq 4000. Expression levels of 22 selected differentially expressed genes (DEG) were assessed by nCounter analysis in an independent series of 71 ccRCCs. A clinicopathological-molecular model for predicting tumor progression was developed and in silico validated in a total of 202 ccRCC patients using the TCGA cohort. A total of 1202 DEGs were found between progressive and non-progressive intermediate/high-risk ccRCC in RNAseq analysis, and seven of the 22 DEGs selected were validated by nCounter. Expression of *HS6ST2*, pT stage, tumor size, and ISUP grade were found to be independent prognostic factors for tumor progression. A risk score generated using these variables was able to distinguish patients at higher risk of tumor progression (HR 7.27; *p* < 0.001), consistent with the results obtained from the TCGA cohort (HR 2.74; *p* < 0.002). In summary, a combined prognostic algorithm was successfully developed and validated. This model may aid physicians to select high-risk patients for adjuvant therapy.

## 1. Introduction

Clear cell renal cell carcinoma (ccRCC) is the most frequent renal tumor, accounting for 80–90% of cases, and has the greatest malignant potential of all renal cell carcinoma subtypes. The mainstay treatment for non-metastatic ccRCC is partial/radical nephrectomy. Despite surgical treatment, approximately between 25 to 50% of these patients will develop local relapse or distant metastases during follow-up [1,2,3].

Several prognostic algorithms have been designed to quantify the likelihood of developing disease progression [4,5]. Pathological stage, tumor size, and the Fuhrman/ International Society of Urological Pathology (ISUP) grading system appear to be the most significant prognostic factors [6,7]. Although current risk models including these variables have managed to classify patients into low-, intermediate-, and high-risk of progression, these are insufficient to accurately predict tumor aggressiveness and prognosis at the individual patient-level as tumor biology might not be entirely considered.

Gene expression profiles have been demonstrated to provide valuable prognostic information in several cancer types [8,9], including ccRCC [10,11]. However, as far as we know, none of the proposed classifiers for ccRCC are currently used in clinical practice, nor validated in intermediate/high-risk patients. These patients exhibit a metastatic potential of over 30% [4] and a high mortality rate of between 20–50% at 5 years [12], and yet no adjuvant strategies are recommended by the European Guidelines [13]. Several clinical trials for adjuvant systemic treatment have already been published. However, most of the disappointing results are due to the poor patient selection criteria based only on clinicopathological characteristics. Optimizing the criteria of patient selection has been revealed as fundamental for obtaining positive outcomes. Hence, it is crucial to identify those patients at highest risk of progression in this specific subset of patients, since they may benefit from a better medical management. 

Here, we examined gene expression profiles in intermediate/high-risk ccRCC to identify prognostic biomarkers and develop a combined prognostic algorithm, including clinicopathological features and molecular biomarkers, to better predict the potential risk of recurrence after surgery.

## 2. Results

### 2.1. Clinical Features of the Cohort

The clinicopathological characteristics of the intermediate/high-risk ccRCC patients and their follow-up information, split by study phases, are listed in Table 1.

Median follow-up of the cohort was 110.76 mo (range 1.1–255.6). During follow-up, 31 (48.43%) patients developed tumor progression. All of them had distant metastasis. Median time to relapse was 12.72 months (range 1–84.84). Twenty-one (67%) of the progressive patients received adjuvant treatment, and 19 (90.5%) remained with stable disease. Thirteen patients (20.3%) died from ccRCC. None of the non-progressive patients received adjuvant treatment. Thirteen patients (20.3%) died from ccRCC.

### 2.2. Biomarker Discovery Phase

**Figure 1:**
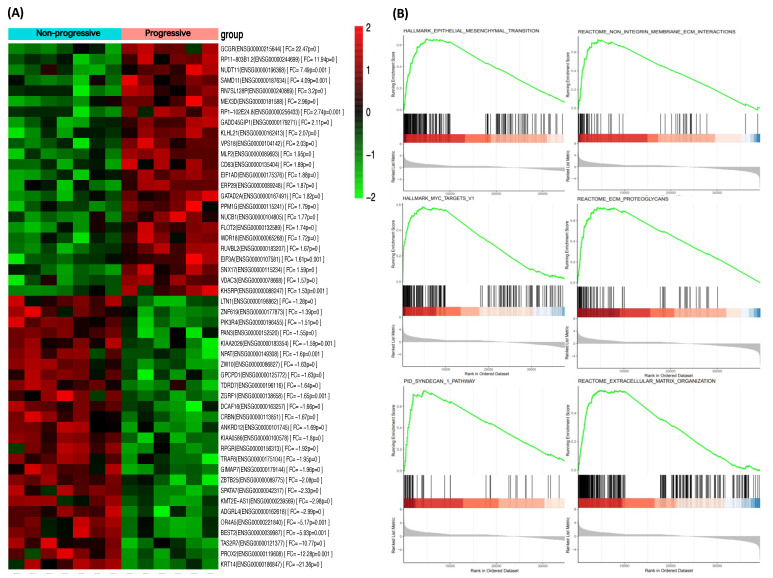
Differentially expressed genes in the discovery phase. (**A**) Heat map displaying the 50 most DEGs between progressive and non-progressive intermediate/high-risk ccRCC patients. Red pixels correspond to up-regulated genes, whereas green pixels indicate down-regulated genes. (**B**) GSEA shows positive correlation of DEGs in pathways involved in tumor progression. Abbreviations: DEGs, Differentially expressed genes. GSEA, Gene set enrichment analysis.

Overall, 1202 genes were identified as differentially expressed between progressive and non-progressive intermediate/high-risk ccRCC patients. Of these, 591 were downregulated and 611 upregulated in progressive compared with non-progressive cases. A heat map based on the most DEGs between the two groups of ccRCC patients is shown in Figure 1A.

Gene set enrichment analysis (GSEA) based on Hallmark, KEGG, and Reactome databases identified that DEGs were positively enriched in pathways, such as the epithelial-mesenchymal transition (EMT), ECM proteoglycans, non-integrin membrane ECM interactions, extracellular matrix organization, MYC targets V1, and PID syndecan 1 pathway, among others (Figure 1B). A complete list of enriched pathways and their target genes is available in Supplementary Materials (Table S1). 

### 2.3. Biomarker Validation Phase

Twenty-two DEGs selected from the previous phase were analyzed by nCounter in an independent cohort of 64 intermediate/high-risk ccRCC samples. Seven (31.8%) genes remained significantly differentially expressed: *DUOX1*, *HS6ST2*, *KRT20*, *RCOR2*, *SFN*, *SSC4D*, and *WNT9A.* All were upregulated in progressive patients. According to IPA, these seven validated DEGs were enriched in organismal injury and abnormalities, renal and urological disease network, and in malignant genitourinary solid tumor diseases, among others. Significant IPA canonical pathways are depicted in Figure S1. The generated network by GeneMANIA shows that there are no direct interactions between the seven significant DEG, although some of them show co-expression (Figure S2).

### 2.4. Survival Analyses

Univariate and multivariate Cox regression analysis of the seven validated DEGs and the six clinicopathological variables showed that *HS6ST2*, pathological stage, tumor size, and ISUP grade were independent prognostic factors of tumor progression (Table 2).

**Figure 2:**
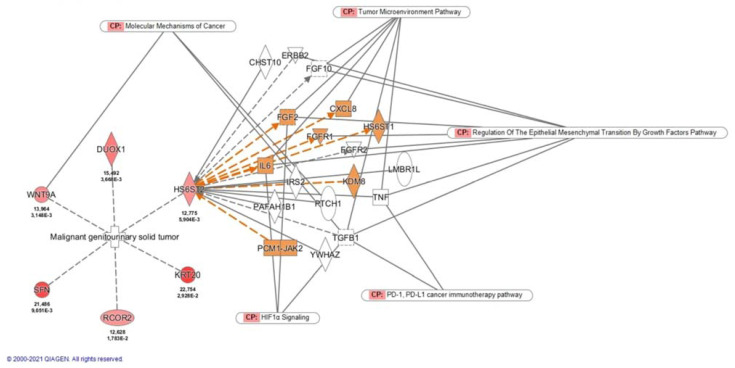
Disease and functions related to the validated genes and *HS6ST2* significant molecular interactions and involved pathways. Abbreviations: CP, Canonical pathways.

Using IPA we found that *HS6ST2* interacts with several molecules involved in different cancer pathways, as illustrated in Figure 2. Moreover, *HS6ST2* was found to be associated with formation, activation, sprouting, and tubulation of vascular endothelial cells. Tumor development and angiogenesis are part of the five top organismal processes related to *HS6ST2*.

### 2.5. Classifier Development Phase

**Figure 3:**
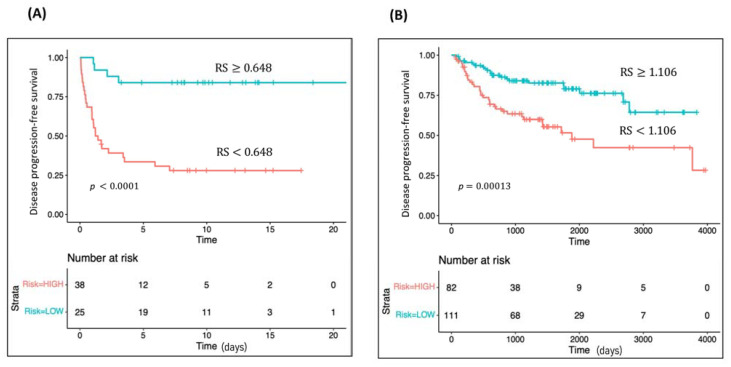
Performance of the combined classifier. Kaplan Meier survival analyses for tumor progression according to the combined classifier (**A**) in our cohort and (**B**) in the TCGA cohort.

The RS for disease progression was calculated for each patient according to a mathematical algorithm containing *HS6ST2* expression values, pathological stage, ISUP grade, and tumor size. A ROC analysis of this combined gene expression-clinicopathological model was performed, and the optimal Youden’s index cut-off value (0.648) was used to classify patients into high- and low-risk groups for tumor progression (Area Under the Curve [AUC] 0.838). Kaplan Meier curve of the combined classifier generated using the selected threshold was able to discriminate two groups with a significantly different probability of tumor progression (hazard ratio (HR) 7.27; *p* < 0.0001) (Figure 3A). Remarkably, the predictive combined model outperformed disease progression prediction of clinicopathological variables, as well as of *HS6ST2* gene expression (Figure S3).

### 2.6. Classifier In Silico Validation 

We evaluated the predictive capability for disease progression of our combined model in 202 intermediate/high-risk ccRCC samples obtained from the TCGA cohort. A RS for disease progression was computed for each patient. A ROC analysis was performed, and the Youden’s index value (1.106) was used as a cut-off. The AUC value of the model was 0.678, showing greater discriminatory ability than clinicopathological variables and gene expression alone (AUC pT = 0.620; AUC tumor size = 0.669; AUC ISUP grade = 0.556; AUC *HS6ST2* = 0.642). The Kaplan Meier curve revealed that patients with a high-risk score have shorter disease-free survival (HR 2.74; *p* < 0.0002) (Figure 3B). Moreover, we carried out a Kaplan Meier sub-analysis using the RS of our combined model to evaluate its performance in the intermediate and high-risk ccRCC samples separately. The combined classifier was found to be statistically significant in the subset of high-risk ccRCC TCGA cohort (HR 2.40; *p* = 0.015). (Figure S4).

## 3. Discussion

Current risk stratification models for non-metastatic ccRCC are based mainly on clinicopathological factors. According to the SSIGN score [5], intermediate and high-risk ccRCC have a disease progression rate of 30 and 70% at 5 years, respectively, and two-thirds of these patients relapse during the first year of follow-up [3]. Several randomized clinical trials (RCT) [14,15] have investigated the benefits of tyrosine kinase inhibitor-based adjuvant therapy in these groups of patients to improve disease progression-free survival (DFS). However, results from these RCT are controversial, as only one of them [14] was positive for its primary endpoint (DFS). The most accepted explanation is that intermediate/high-risk ccRCC involves a heterogeneous group of tumors that do not share neither clinicopathological nor molecular characteristics [16]. Recently, interim data from the first phase-III adjuvant immunotherapy trial [17] has shown an improvement in DFS using an anti-PD-1 agent; however, to date, several adjuvant immunotherapy trials are ongoing, and the dissimilar patient selection criteria will most probably translate into important survival differences [18]. This emphasizes the critical need for more reliable and individualized prognostic markers to select ccRCC patients at the highest risk of recurrence to receive adjuvant treatment or to avoid overtreatment and toxic side effects in those with low progression risk.

The identification of molecular markers and the development of multigene classifiers have already been shown to considerably increase the predictive power of the clinical parameters. In this study, we determined a set of DEGs related to progression in intermediate/high-risk ccRCC using RNAseq. GSEA corroborates that the DEGs identified participate mainly in canonical pathways related to carcinogenetic, cell-cycle, and metabolic processes. To the best of our knowledge, our study is the first to report differential gene expression patterns between progressive and non-progressive intermediate/high-risk ccRCC. Validation of a subset of DEGs in an independent series allowed us to select potential prognostic biomarkers.

Our study indicates that *HS6ST2* may serve as a useful prognostic marker among intermediate/high-risk ccRCCs to predict disease progression. Furthermore, we developed and validated, in an independent cohort, a model including *HS6ST2* expression levels and three clinicopathological factors which significantly improves the prognostic accuracy upon clinicopathological characteristics for discriminating patients at higher risk of relapse. Our prognostic model is able to classify intermediate/high-risk ccRCC patients into two groups of patients with a different probability of tumor progression which may help physicians to tailor disease management, individualize follow-up, and serve as a tool to improve patient selection for adjuvant treatment in clinical trials. Unfortunately, most of the reported ccRCC gene-based classifiers include all clinical stages of ccRCC; thus, the classifiers’ performance cannot be comparable [10,11]. Only Wu et al. [19], using mRNA expression data from GSE53757 and TCGA databases, evaluated stage III ccRCC prognosis. However, contrary to our study, they analyzed gene expression differences between normal tissue and stage III ccRCC. Furthermore, their model was focused on overall survival, not on disease progression, as in ours.

*HS6ST2* (heparan sulfate D-glucosaminyl 6-O-sulfotransferase-2) encodes an enzyme that catalyzes the transfer of sulfate groups in heparan sulfate proteoglycans (HSPGs). HSPGs participate in the regulation of numerous signaling pathways by interacting with various heparin-binding ligands and activating cytokines to influence cell growth, differentiation, adhesion, and migration [20,21]. In vitro and in vivo evidence indicates that *HS6ST2* is essential for vascular development [22,23] and plays an important role in tumor angiogenesis due to its interactions with several angiogenic growth factors, including FGF2, VEGF, IL8, and IL6, among others [24,25]. Furthermore, evidence suggests that *HS6ST2* is also associated with EMT by interacting with TGF-beta, HIF-1, and estrogen/GPER pathways [24,26,27,28,29], activation of T lymphocytes, and indirectly related with the PD-1, PDL-1 cancer immunotherapy pathway. Interesting, immune-oncology checkpoint inhibitors are being investigated as a potential first line adjuvant therapy after nephrectomy in non-metastatic ccRCC. Numerous studies have described that inhibition of *HS6ST2* in tumor cells impairs cell migration, invasion, and tumor angiogenesis and may reverse EMT [20]. Unsurprisingly, reduction of *HS6ST2* expression has been investigated as a potential target for future therapies [30]. 

In line with our findings, overexpression of *HS6ST2* has been found to be a poor prognostic factor in several malignant tumors [20,24,30,31,32,33]. Interestingly, *HS6ST2* has been recently described as part of a novel 10 glycolysis-related gene signature to predict overall survival in ccRCC [34]. Liep et al. [35] found that overexpression of miR-145-5p and miR-141-3p could inhibit the migration and invasion of RCC cells by decreasing *HS6ST2* expression in cellular transfection experiments. Although more studies are required to better comprehend the involvement of this gene in cancer biology, its relationship with tumor development and angiogenesis have important clinical implications. VEGF-target therapies have been the first-line standard of care for metastatic ccRCC and still have a relevant role in metastatic ccRCC as a partner with anti-PD1/L1 immunotherapy in first-line treatment or in monotherapy at progression, due to the natural chemoresistance and radioresistance shown by tumoral renal cells.

Our study has multiple strengths. The first is that we assessed intermediate/high-risk ccRCC patients in a multicenter study. Despite it being well known that these patients have a higher risk of recurrence, our classifier may contribute to determine which of them could most benefit from receiving the new targeted adjuvant treatment. Secondly, RNA was isolated from FFPE ccRCC tissue obtained in routine practice, and the technology used to quantify gene expression is highly cost-effective, facilitating its implementation in the clinical setting. Thirdly, our non-progressive ccRCC patients had a long-term follow-up to avoid misclassification. Lastly, the researchers participating in this study were blinded to all clinical information, and gene expression was matched to clinical data only after all patient cases had been processed.

Nevertheless, we acknowledge some study limitations. First, the retrospective design and the small sample used in the validation phase. Secondly, the biomarker selected may not apply to low-risk tumors and should not be generalizable to all patients with ccRCC. Finally, external validation in the TCGA ccRCC samples revealed that our combined classifier has a good performance predicting disease progression; nevertheless, further experimental validation in larger cohorts is required.

## 4. Materials and Methods

### 4.1. Patients

A multicenter study in which a total of 84 non-metastatic intermediate/high-risk ccRCCs, as defined by the Mayo Clinic Stage, Size, Grade, and Necrosis (SSIGN) score [5], who underwent partial or radical nephrectomy between 2000 and 2012 in two different centers (Hospital Clinic of Barcelona, Barcelona, Spain, and Oncologic Institute of Valencia, Valencia, Spain), were retrospectively included.

**Figure 4:**
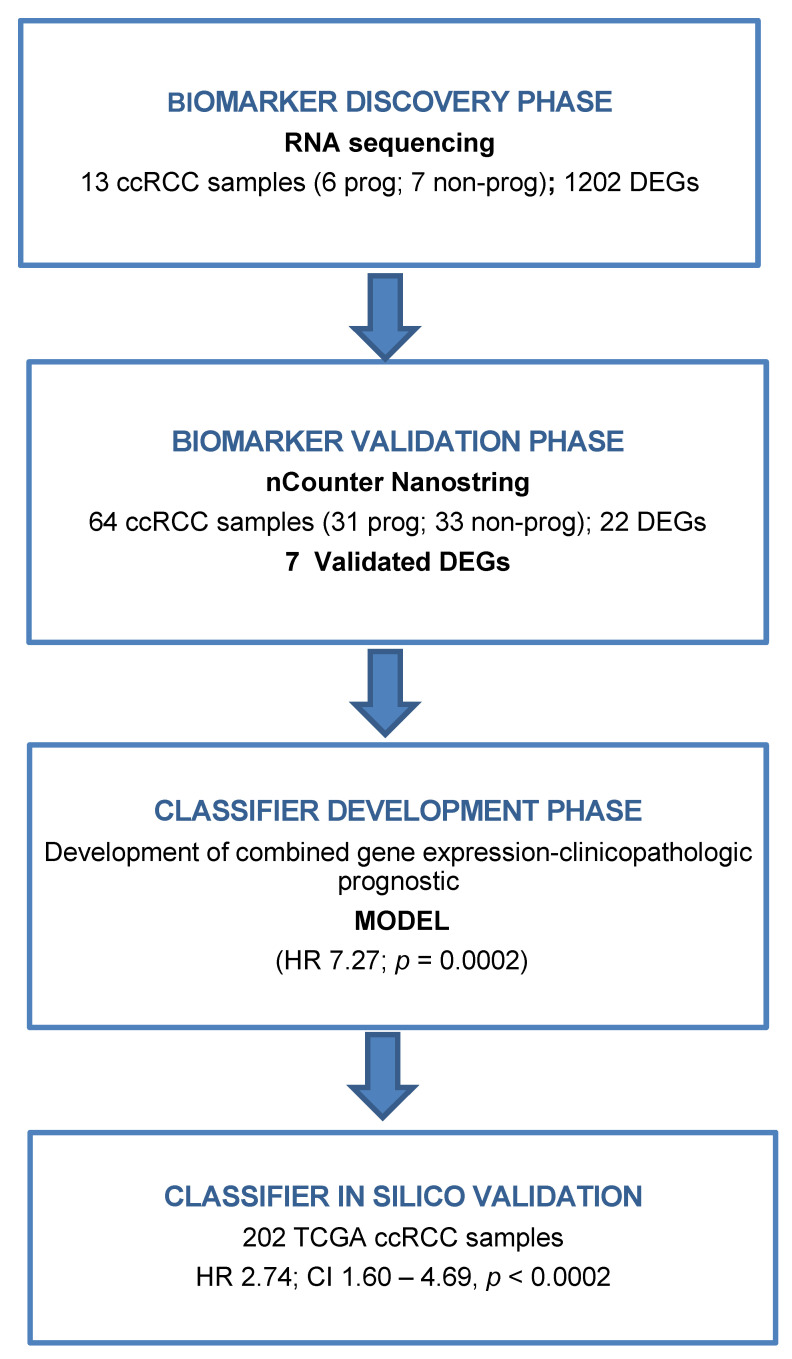
Study outline. Tissue samples were obtained from a total of 77 patients with intermediate/high-risk ccRCC. Samples were split into a biomarker discovery (*N* = 13) and validation (*N* = 64) phase. Transcripts differentially expressed between progressive and non-progressive intermediate/high-risk ccRCCs were first identified in the discovery phase using RNAseq. Twenty-two DEGs were selected for validation in an independent set of 64 tissue samples using nCounter Elements (Nanostring). A prognostic model was generated using gene expression and clinical data in the classifier development phase. Finally, the prognostic model was in silico validated using a TCGA cohort. Abbreviations: ccRCC, Clear cell renal cell carcinoma, DEGs, Differentially expressed genes.

This study was split into four-stage approach with an initial biomarker discovery phase, a biomarker validation phase, a classifier development phase, and, lastly, the in silico external validation using the TCGA cohort [36,37] (Figure 4). Initial discovery phase included 13 ccRCC patients from Hospital Clinic of Barcelona, six progressive and seven non-progressive. Biomarker validation phase comprises 71 ccRCC patients from the Oncologic Institute of Valencia (IVO). From this, 64 ccRCC patients were eventually included in the study, 31 progressive and 33 non-progressive (Table 1). The remaining seven patients were discarded because of low count values in Nanostring analysis. Tissue samples were obtained under institutional review board-approved protocols (HBC/2016/0333 and 2017-59-BIOBANCOFIVO-15-2017).

All patients were followed-up postoperatively according to the European Urology guidelines [13]. Briefly, CT scans were performed at the 3rd month after surgery, every 6-months for the first 3 years, annually until 5 years, and biannually thereafter. Tumors were considered as progressive when local relapse or distant metastasis were developed during the follow-up. Non-progressive patients had a minimum follow-up of 10 years.

### 4.2. Tissue Specimens and RNA Isolation

Formalin-fixed paraffin-embedded (FFPE) tissue blocks were reviewed. RNA was isolated from FFPE specimens (total thickness 80 µm) using the RecoverAll™ Total Nucleic Acid Isolation kit for FFPE (Ambion, Inc., Austin, TX, USA) following manufacturer’s instructions. RNA was quantified by spectrophotometric analysis at 260 nm (NanoDrop Technologies, Wilmington, DE, USA). 

### 4.3. Biomarker Discovery Phase—RNA Sequencing

#### 4.3.1. Library Preparation and Sequencing Method

RNA from 13 selected ccRCC samples was processed for library preparation using the TruSeq^®^ RNA Access Library Preparation Kit (Illumina, San Diego, CA, USA) that allows generating libraries starting from degraded RNA. Briefly, cDNA strands were synthetized from input RNA in order to be adaptor-tagged, labeled, and amplified. cDNA was then pooled and enriched by a double step of probe hybridization. The enriched targets were captured by streptavidin labeled beads, cleaned up, and amplified to obtain the final multiplexed libraries. The libraries were then sequenced on an Illumina HiSeq^®^ 4000 platform (Illumina, San Diego, CA, USA).

#### 4.3.2. Read Alignment and Differential Gene Expression Analysis

Paired-end RNA-Seq FASTQ files were trimmed from a 3′ end to a fixed length based on the Phred quality score (trimmed if score fell below 20, with a minimum read length of 25) [38]. Trimmed RNA-seq reads were aligned to the GRCh38 reference genome with STAR [39], and gene counts were determined using quantMode GeneCounts. Trimmed reads were then aligned using STAR. We used limma-voom transformation and cyclic-loess to normalize the non-biological variability. An assessment of differential expression between groups was evaluated using moderated *t*-statistics [40]. Significant DEGs in progressive relative to non-progressive patients were identified based on an adjusted *p* value of <0.05. The heatmap and statistical analyses were performed using R statistical package (v3.3.2). Gene set enrichment analysis (GSEA) was performed using GSEA2-2.2.0 software for testing specific gene sets based on Hallmark, Kyoto Encyclopedia of Genes and Genomes (KEGG), and Reactome pathway databases [41]. RNAseq files and clinical information were deposited into Gene Expression Omnibus (GEO) with the accession number GSE175648.

### 4.4. Biomarker Validation Phase

#### 4.4.1. nCounter Elements Gene Expression Analysis

The 22 differentially expressed genes (DEGs) with a higher fold change were selected from the discovery phase and validated in an independent cohort of 71 ccRCC patients. nCounter Elements Analysis System (NanoString Technologies, Seattle, WA, USA) was used to quantify gene expression from these 22 targets and two housekeeping (*PPIA* and *TBP*) genes [42]. Briefly, probes were hybridized with the Elements TagSet and 250 ng of RNA samples for 21 h at 67 °C in a Thermal Cycler. Thereafter, samples were purified in the nCounter Prep Station to remove unligated probes. Expression data were collected using the nCounter Digital analyzer. Those counts with values ≤ 10 were excluded from the analysis, and those genes (*n* = 14) expressed in less than 80% of the samples were discarded for further examinations. Furthermore, those samples (*n* = 7) with less than 80% of expressed genes were excluded from further analysis. Empty input values for each gene were inputted to the minimum count value for that gene. Gene expression normalization was performed using the *PPIA* housekeeping gene since *TBP* was discarded because of its lack of expression in more than 20% of samples.

#### 4.4.2. Survival Analysis

Cox stepwise regression analysis was used on the established clinical prognostic factors of ccRCC (pT stage, pN status, ISUP grade, tumor size, necrosis, vascular, and perirenal fat invasion) and the seven DEGs to investigate their influence on tumor progression. Statistical significance was defined at a *p* value of 0.05. 

### 4.5. Classifier Development Phase

After establishing the multivariate model, a risk score (RS) for the variables of the model was calculated for each patient. RS was subjected to a Receiver Operating Characteristics (ROC) curve analysis to choose the most appropriate threshold for predicting tumor progression. Thereafter, Kaplan-Meier curves were generated using the selected cut-off point and compared according to the log-rank test. 

### 4.6. Pathway Enrichment Analysis

Ingenuity Pathway Analysis (IPA) software was used to identify interactions and networks between significant DEGs, possible altered canonical pathways, regulators, diseases, and functions based on direct/indirect and experimental targets.

### 4.7. Classifier In Silico Validation

The Cancer Genome Atlas (TCGA) gene expression dataset was obtained from portal (https://firebrowse.org) (accessed on 8 October 2021) [36], and TCGA clinical data were downloaded from the portal (https://www.sciencedirect.com/science/article/pii/S0092867418302290?via%3Dihub#app2) (accessed on 8 October 2021) [37]. After selecting samples matching our selection criteria and excluding patients without survival status or missing clinical data, a total of 202 ccRCCs were selected. Level 3 RNAseq expression data from ccRCC samples by the Illumina HiSeq2000 RNA sequencing platform, and corresponding clinical data were downloaded from the aforementioned websites.

## 5. Conclusions

A novel prognostic algorithm based on gene expression and clinicopathological factors was successfully developed and validated in the TCGA cohort. This model is able to identify a subset of intermediate/high-risk ccRCC patients with a higher risk of tumor progression and may contribute to identify those patients that would most benefit from closer surveillance and adjuvant treatment. 

## Figures and Tables

**Table 1:** Demographic and pathological characteristics of enrolled patients.

Clinicopathological Characteristics	Discovery Phase Hospital Clinic Barcelona (*n* = 13)	Validation Phase Institute Valenciano of Oncology (*n* = 64)
Gender		
Male	9 (69.2)	49 (76.6)
Female	4 (30.8)	15 (23.4)
Age at diagnosis (yr)	54.85 (36–81)	58.6 (35–87)
Pathological tumor size (cm)	8.2 (2.5–14)	8 (3.1–24)
ISUP		
ISUP 1	-	4 (6.3)
ISUP 2	2 (15.4)	20 (31.3)
ISUP 3	6 (46.2)	31 (48.4)
ISUP 4	5 (38.4)	9 (14)
Tumor stage		
pT1	5 (38.4)	9 (14)
pT2	5 (38.4)	16 (25)
pT3	2 (15.4)	36 (56.3)
pT4	1 (7.8)	3 (4.7)
N stage		
N0/x	11 (84.6)	58 (90.6)
N1	2 (15.4)	6 (9.4)
Perirenal fat invasion	3 (23.1)	40 (62.5)
Vascular invasion	2 (15.4)	10 (15.6)
Necrosis	1 (7.8)	24 (37.5)
SSIGN score		
Intermediate risk	7 (53.8)	39 (60.9)
High risk	6 (46.2)	25 (39.1)

( ) Range or %.

**Table 2:** Univariate and multivariate Cox regression analysis of statistically significant genetic and clinical variables in the validation set.

Genes	Univariate			Multivariate		
	*p*	95% CI	HR	*p*	95% CI	HR
***DUOX1***	<0.001	1.404–2.751	1.965			
***HS6ST2***	0.001	2.084–14.157	5.432	<0.001	2.710–14.880	6.35
***KRT20***	<0.001	1.419–2.926	2.037			
***RCOR2***	<0.001	2.417–16.627	6.340			
***SFN***	<0.001	1.806–5.647	3.194			
***SSC4D***	0.001	1.982–14.831	5.422			
***WNT9A***	0.002	1.600–7.475	3.459			
**pT Stage**	<0.001	1.610–5.362	2.939	0.016	1.150–4.090	2.17
**Tumor size**	<0.001	1.068–1.246	1.154	0.018	1.020–1.230	1.12
**ISUP**	0.045	1.010–2.533	1.599	0.021	1.100–3.370	1.93

## Data Availability

The data presented in this study are available in the article and supplementary material. RNAseq files and clinical information were deposited into Gene Expression Omnibus (GEO) with the accession number GSE175648. Further details can be obtained on request from the corresponding author.

## Supplementary Materials

The following are available online at https://www.mdpi.com/article/10.3390/cancers13246338/s1, Figure S1. Significant canonical pathways and overlapping network for the seven genes validated, Figure S2. Gene-gene interaction network for the 7 significant differentially expressed genes, Figure S3. Performance of the combined classifier. ROC curves for predicting tumor progression after surgery according to the combined classifier, gene expression, and clinicopathological variables, Figure S4. Kaplan Meier survival analyses for tumor progression according to the combined classifier (A) in high-risk TCGA Cohort and (B) in the intermediate-risk TCGA cohort, Table S1: Enriched pathways and their target genes from the DEG in the RNAseq analysis.
