# Supplementary figures and images for: Clinicopathological and Molecular Prognostic Classifier for Intermediate/High-Risk Clear Cell Renal Cell Carcinoma
Source: Cancers (Basel). 2021 Dec 17;13(24):6338. doi: 10.3390/cancers13246338 (PMC8699125; doi:10.3390/cancers13246338)

Supplementary Figure S1.

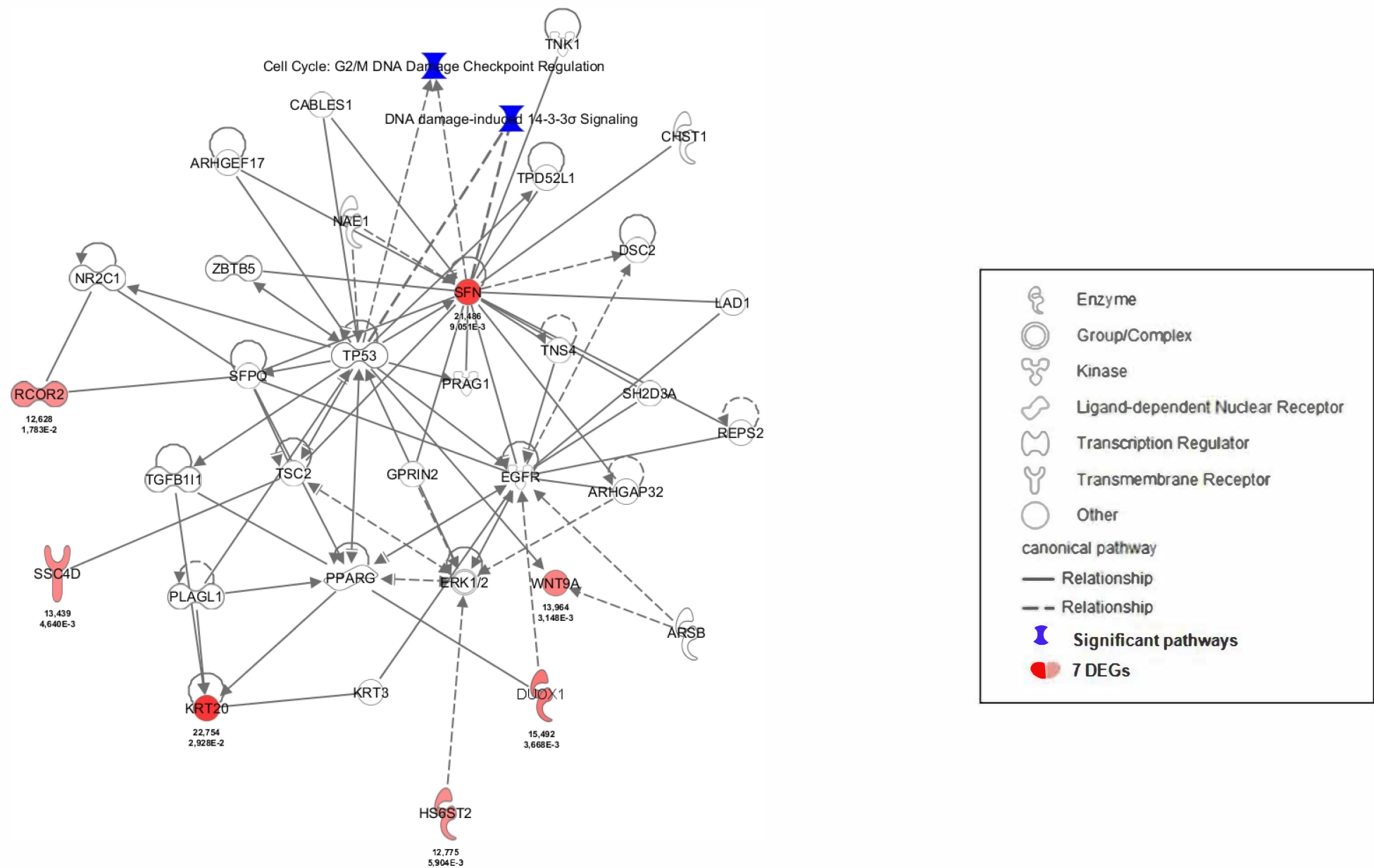

Supplement: Supplementary file 1 [file cancers-13-06338-s001.zip › Figure S1.pdf]

Supplementary Figure S2.

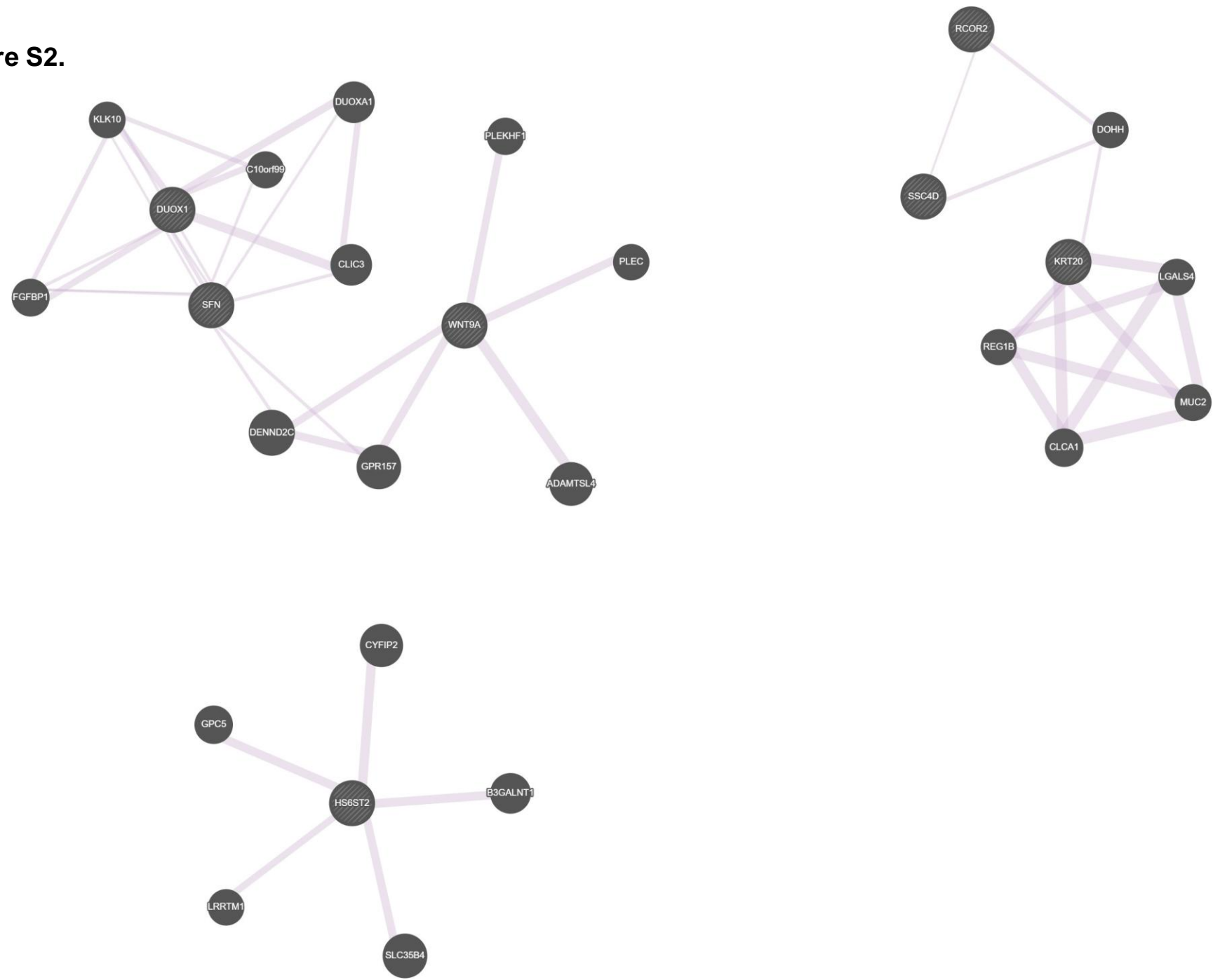

Supplement: Supplementary file 1 [file cancers-13-06338-s001.zip › Figure S2.pdf]

**Supplementary Figure S3**

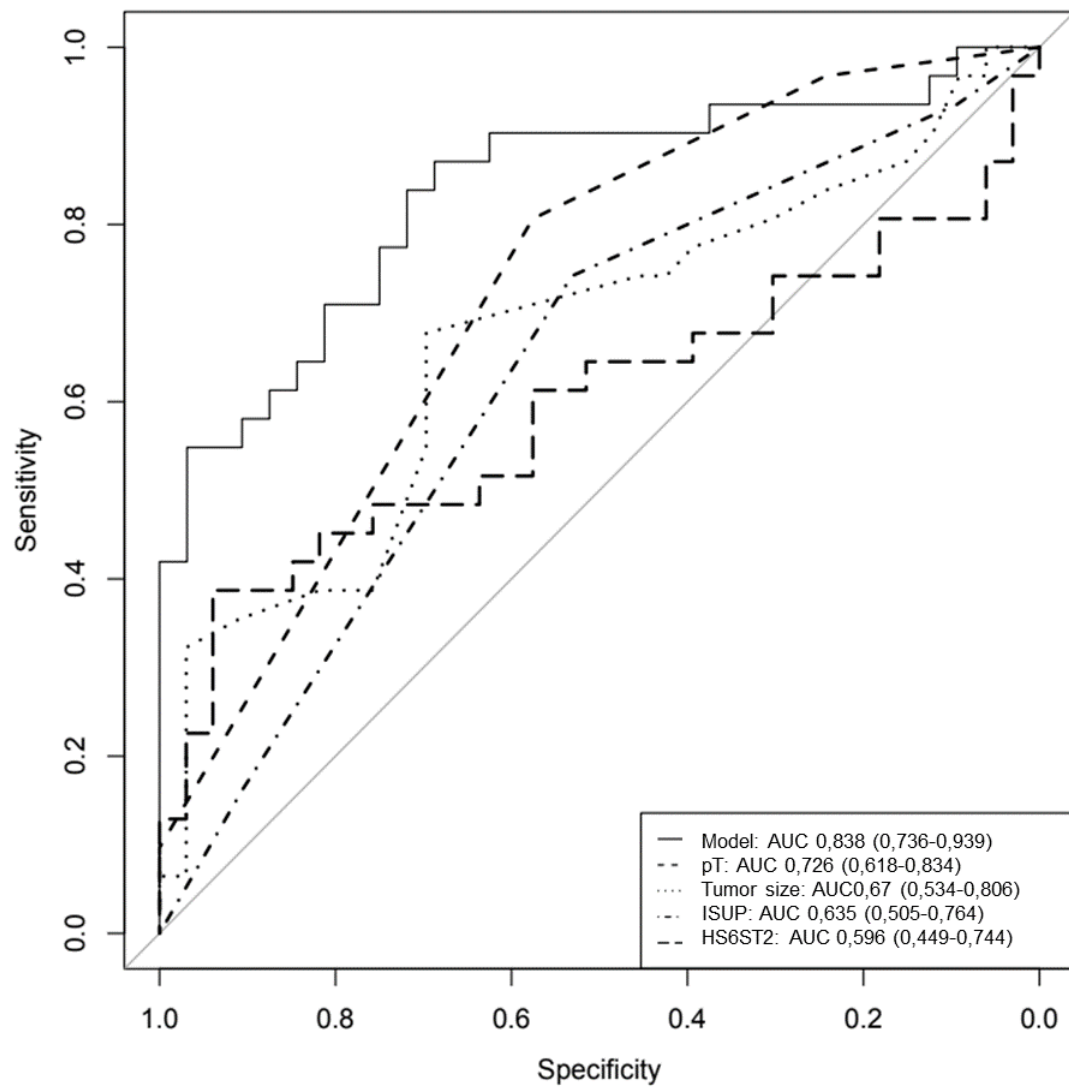

Supplement: Supplementary file 1 [file cancers-13-06338-s001.zip › Figure S3.pdf]

Supplementary Figure S4.

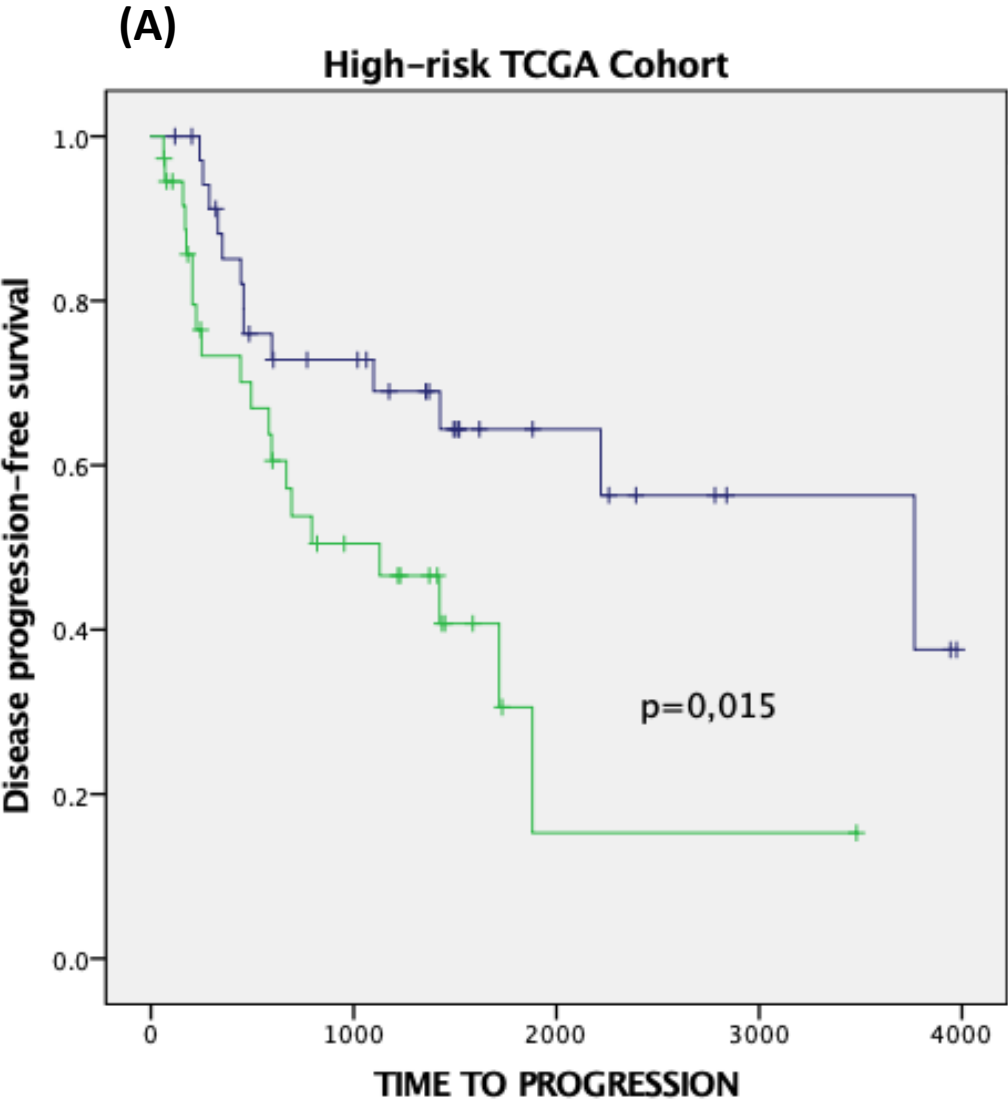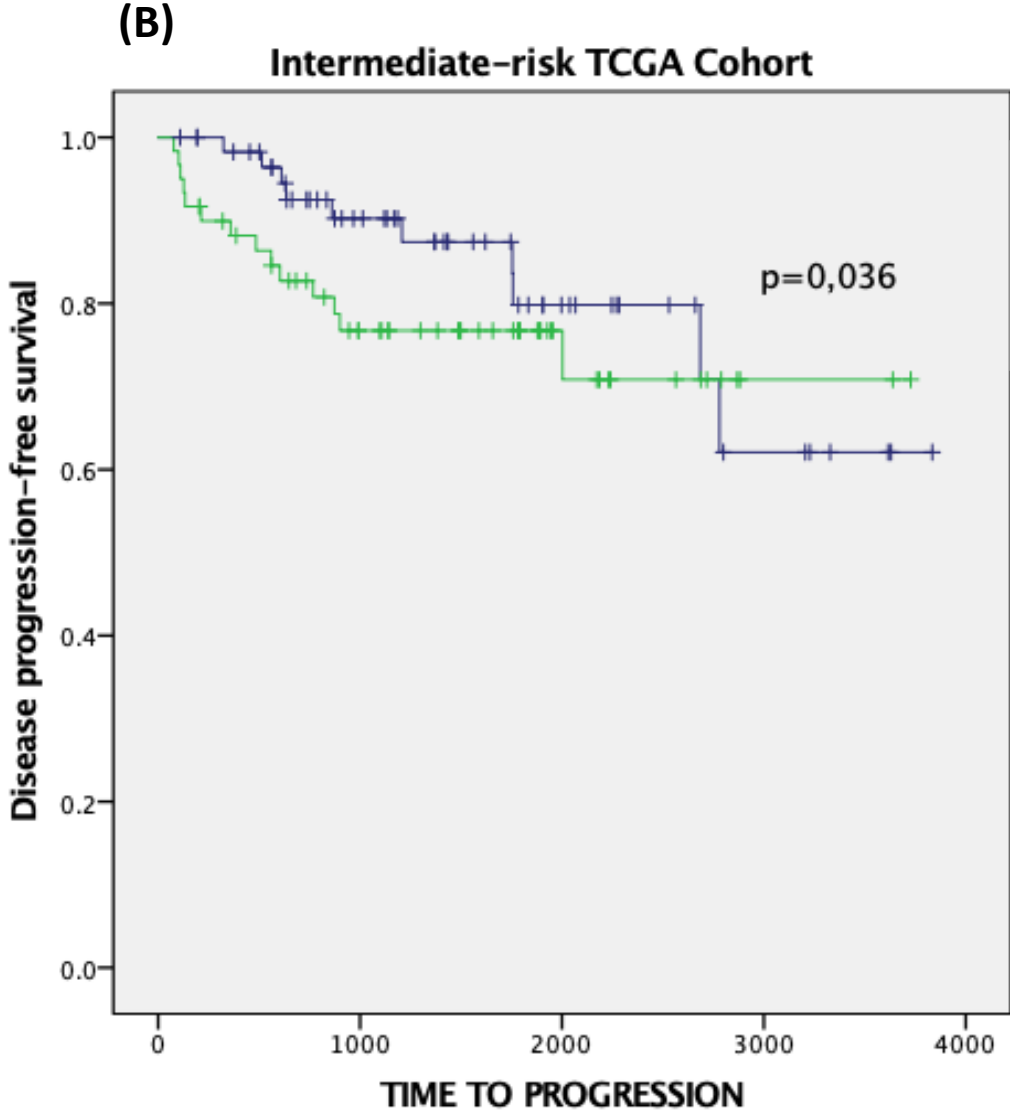

Supplement: Supplementary file 1 [file cancers-13-06338-s001.zip › Figure S4.pdf]
